# Careful Selection of Reference Genes Is Required for Reliable Performance of RT-qPCR in Human Normal and Cancer Cell Lines

**DOI:** 10.1371/journal.pone.0059180

**Published:** 2013-03-15

**Authors:** Francis Jacob, Rea Guertler, Stephanie Naim, Sheri Nixdorf, André Fedier, Neville F. Hacker, Viola Heinzelmann-Schwarz

**Affiliations:** 1 Gynecological Research, Department of Biomedicine, University Hospital Basel, Basel, Switzerland; 2 Ovarian Cancer Group, Adult Cancer Program, Lowy Cancer Research Centre, University of New South Wales, Sydney, Australia; 3 Gynaecological Cancer Centre, Royal Hospital for Women, Sydney, Australia; Institute for Systems Biology, United States of America

## Abstract

Reverse Transcription - quantitative Polymerase Chain Reaction (RT-qPCR) is a standard technique in most laboratories. The selection of reference genes is essential for data normalization and the selection of suitable reference genes remains critical. Our aim was to 1) review the literature since implementation of the MIQE guidelines in order to identify the degree of acceptance; 2) compare various algorithms in their expression stability; 3) identify a set of suitable and most reliable reference genes for a variety of human cancer cell lines. A PubMed database review was performed and publications since 2009 were selected. Twelve putative reference genes were profiled in normal and various cancer cell lines (n = 25) using 2-step RT-qPCR. Investigated reference genes were ranked according to their expression stability by five algorithms (geNorm, Normfinder, BestKeeper, comparative ΔCt, and RefFinder). Our review revealed 37 publications, with two thirds patient samples and one third cell lines. qPCR efficiency was given in 68.4% of all publications, but only 28.9% of all studies provided RNA/cDNA amount and standard curves. GeNorm and Normfinder algorithms were used in 60.5% in combination. In our selection of 25 cancer cell lines, we identified *HSPCB*, *RRN18S*, and *RPS13* as the most stable expressed reference genes. In the subset of ovarian cancer cell lines, the reference genes were *PPIA*, *RPS13* and *SDHA*, clearly demonstrating the necessity to select genes depending on the research focus. Moreover, a cohort of at least three suitable reference genes needs to be established in advance to the experiments, according to the guidelines. For establishing a set of reference genes for gene normalization we recommend the use of ideally three reference genes selected by at least three stability algorithms. The unfortunate lack of compliance to the MIQE guidelines reflects that these need to be further established in the research community.

## Introduction

Reverse Transcription (RT) - quantitative Polymerase Chain Reaction (qPCR) has become a versatile technique to examine expression changes of one or more genes of interest in various pathological states. Due to its specificity, sensitivity, simplicity, costs and high-throughput, RT-qPCR offers a broad range of advantages over standard methods such as Northern blot and semi-quantitative PCR. Therefore it has become the most emerging tool for absolute and relative quantification of mRNA transcription levels [1]. RT-qPCR is a robust assay that uses well-established chemistry and data analysis and is therefore superior to techniques such as Southern blotting or DNA sequencing [2]. Despite its broad acceptance, there are inconsistencies in the use of total RNA extraction methods, RNA quantity per reaction [3], RNA integrity assessments [4], qPCR master mixes and in the various manufacturers of the reverse transcription kits. In addition, the use of different qPCR detection methods such as dye- or probe-based systems broadens the spectrum of potential applications. In order to overcome potential difficulties and to achieve consensus in the performance and analysis of quantitative PCR experiments, the MIQE (Minimum Information for Publication of Quantitative Real-Time PCR Experiments) guidelines were introduced in 2009, enhancing the experimental design [5]. In addition, the application of these guidelines will deliver better and reproducible results by reporting parameters such as RNA integrity, reaction volume, cDNA/RNA concentration, or calibration curves [6].

Target gene normalization is usually achieved using reference genes to compensate intra- and inter-kinetic RT-qPCR [7]. Several mathematical approaches have been published that deliver suitable reference genes with the lowest variation and with high stability across the biological samples. The four most commonly used approaches are: (1) NormFinder algorithm [8] which is a statistical model that estimates the overall variation of gene expression for each candidate reference gene and delivers a stability value. This value is related to the systematic error of each candidate gene. (2) GeNorm [9] calculates a gene stability measure for each candidate gene. The reference gene with the lowest stability (M) is removed from further analysis, and M values are repeatedly calculated until the most stable reference gene is left. (3) BestKeeper, a Microsoft^®^ Excel-based tool, uses pair-wise correlations [10]; and (4) the comparative delta Ct (based on the nomenclature and MIQE guidelines: the quantification cycle (Cq) is preferred to the threshold cycle (Ct), both describing the fractional PCR cycle used for quantification) method ranks the stability of candidate genes according to repeatability of gene expression differences [11]. None of the introduced algorithms seems to be optimal and the researcher always has to choose which reference gene to use and how to identify. This may also affect the interpretation of RT-qPCR results.

Numerous putative reference genes have been reported for a wide variety of human tissues, for human cell lines, and for cell cultures under different experimental conditions such as drug treatments or environmental factors. However, no reference genes suitable for the analysis of human cancer cell lines originated from gynecological cancers and colon cancers have yet been described.

The primary aim of this study was to identify the most stable reference genes suitable for the study of normal and cancer cell lines of ovarian or colon origin by profiling a set of 12 putative reference genes and for the first time applying five stability algorithms to estimate the influence of the bioinformatical data analysis. We were also interested to see how different manufacturers of reverse transcriptase kits influence the variation of reference gene expression. In addition, a review of the current literature was performed in order to assess the compliance with the MIQE guidelines in the performance of RT-qPCR reported since their introduction.

## Materials and Methods

### Literature Review

A PubMed (http://www.ncbi.nlm.nih.gov/sites/entrez) database review on the use of putative reference genes in RT-qPCR was performed. Publications available between January 2009 and April 2012 were considered and identified using the key words: ‘reference genes’ OR ‘housekeeping genes’ AND ‘RT-qPCR’ OR ‘quantitative PCR’. Reference lists from selected publications were also considered for inclusion of potentially relevant articles. Publications not written in English and those reporting RT-qPCR performed on less than five reference genes were excluded. Only publications with human samples were included. Publications were evaluated according to the year of publication, the type and amount of samples, the number of reference genes, the methods used to determine RNA integrity, the amount of total RNA initially utilized for RT and/or qPCR, the number of replicates in qPCR, the details on serial dilutions for calibration curve and efficiency, the utilized qPCR chemistry (SYBRgreen or TaqMan), and the mathematical approaches (computing algorithms) for reference gene expression stability measurement.

### Cell Culture

In this study 2 normal and 23 cancer cell lines of various origins were used. The cell lines were derived from ATCC (www.atcc.org) or were a gift from the Garvan Institute of Medical Research, Sydney, Australia. Written informed ethical consent was granted (HREC 08/09/17/3.02, to VHS.). A detailed listing of the cell lines and the culture conditions are given as supplementary data (Table S1). All cell cultures were maintained at 37°C in 5% CO_2_. Cultures were free of mycoplasma, as determined by qualitative PCR using VenorGeM^®^ Mycoplasma Detection Kit (Biocene Pty Ltd, Rozelle, Australia).

### RNA Extraction and Integrity

To examine the expression of the 12 putative reference genes in each of these cell lines, 1×10^5^ cells were grown in 6-well plates (NUNC, Thermo Fisher Scientific, Roskilde, Denmark) to a confluency of 70–90%. Cells were then washed twice with sterile saline and total RNA was extracted using the NucleoSpin RNAII kit (MACHEREY&NAGEL, Scientifix Pty Ltd, Clayton, Australia). For this cells were lysed by adding lysis buffer directly onto the cells. RNA extraction including DNase treatment was carried out according to the manufacturer’s protocol. RNA was eluted in 60 µl RNase free water and RNA concentration measured using NanoDrop ND-1000 spectrophotometer (Thermo Fisher Scientific, Roskilde, Denmark). The integrity of RNA samples was confirmed by agarose gel electrophoresis on 1.0% agarose gel containing GelRed™ (Biotium, Inc., Hayward, CA, USA). RNA samples without indication of degradation were further assessed on an RNA 6000 Nano chip using the Agilent 2100 electrophoresis Bioanalyzer (The Ramaciotti Centre for Gene Functional Analysis, University of New South Wales, Sydney, NSW, Australia).

### Reverse Transcription of mRNA

Total RNA (1 µg) was reverse-transcribed using the iScript Reverse Transcription Supermix for RT-qPCR (#170–8840, MMLV-based RTase, RNaseH^+^, Bio-Rad Laboratories (Pacific) Pty Ltd, Gladesville, Australia) in a total volume of 20 µl according to the manufacturer’s instructions. The Supermix contained both oligo dT and random primers to obtain a maximum number of cDNA transcripts. The reaction mixture was incubated at 25°C for 5 min for priming, then at 42°C for 30 min for reverse transcription, and finally at 85°C for 5 min for reverse transcriptase inactivation. The complementary DNA (cDNA) was stored at −20°C until further use.

In addition to BioRad, we included two other manufactures (Takara and Bioline) to investigate its influence on the performance of the reverse transcription. We reverse transcribed total RNA (1 µg) by six reverse transcriptions (Takara and Bioline) as follows: Reaction mixture was prepared using BluePrint™ 1^st^ strand cDNA synthesis kit (#6115A, MMLV-based RTase, RNaseH^+^, Takara Bio Inc., Scientifix Pty Ltd, Clayton, Australia) in a total volume of 20 µl containing random 6 mers, oligo dT or both in equal molarity. A final volume of 10 µl containing primer, dNTP mixture and total RNA was incubated at 65°C for 5 min and immediately cooled down. Then 5× BluePrint^TM^ 1^st^ strand buffer, recombinant RNase inhibitor and BluePrint^TM^ RTase was added to a final volume of 20 µl. Final reverse transcription was incubated at 30°C for 10 min, then at 42°C for 60 min followed by inactivation at 95°C for 5 min. Reverse transcription using Bioline’s Tetro cDNA synthesis kit (#BIO-65042, MMLV-based RTase, RNaseH^+^, Bioline (Aust) Pty Ltd, Alexandria, Australia) was performed as follows: a final volume of 10 µl containing primer (random 6 mers or oligo dT or both in equal molarity), dNTP mixture and RNA was incubated at 70°C for 5 min, followed by adding 5× RT buffer and Ribosafe RNase inhibitor up to 20 µl. Final reaction mixture was incubated at 45°C for 30 min and terminated at 85°C for 5 min.

### Quantitative Polymerase Chain Reaction (qPCR)

Quantitative PCR was performed on 12 putative reference genes. Their characteristics including name, accession number, chromosome localization, product length, and the respective forward and reverse primers are summarized in Table 1. The reference genes and primer sequences (purchased from Sigma-Aldrich Pty. Ltd, Castle Hill, Australia) were selected based on previous databases and on publications reporting stable gene expression profiles [8], [9], [12]–[15]. Primer sequences were also cross-checked using web-based tool *in-silico* PCR (http://genome.ucsc.edu/cgi-bin/hgPcr) at the human genome browser at UCSC [16] against gene and genomic targets.

**Table 1:** Details of reference genes, primers and amplicons for 12 investigated genes.

Genesymbol	Title	Accessionnumber	ChromosomalLocalization	Forward andReverse Primer	Product(bp)	Intronspanning
***GAPDH***	Glyceraldehyd-3-phosphat-Dehydrogenase	NM_002046	12p13.31	5′-CGACAGTCAGCCGCATCTT-3′ 5′-CCCCATGGTGTCTGAGCG-3′	63	Yes
***RPII (POLR2A)***	polymerase (RNA) II (DNA directed)polypeptide A	NM_000937	17p13.1	5′-GCACCACGTCCAATGACAT-3′ 5′-GTGCGGCTGCTTCCATAA-3′	267	Yes
***TBP***	TATA box binding protein	NM_003194	6q27	5′-TGCACAGGAGCCAAGAGTGAA-3′ 5′-CACATCACAGCTCCCCACCA-3′	132	Yes
***PPIA***	Peptidylprolyl isomerase A (cyclophilin A)	NM_021130	7p13	5′-AGACAAGGTCCCAAAGAC-3′ 5′-ACCACCCTGACACATAAA-3′	118	Yes
***GUSB***	Beta glucuronidase	NM_000181	7q21.11	5′-AGCCAGTTCCTCATCAATGG-3′ 5′-GGTAGTGGCTGGTACGGAAA-3′	160	Yes
***HSPCB***	Heat shock protein 90kDa alpha (cytosolic)	NM_007355	6p12	5′-TCTGGGTATCGGAAAGCAAGCC-3′ 5′-GTGCACTTCCTCAGGCATCTTG-3′	80	Yes
***YWHAZ***	Tyrosine 3-monooxygenase/ tryptophan5-monooxygenase activation protein,zeta polypetide	NM_003406	8q23.1	5′-ACTTTTGGTACATTGTGGCTTCAA-3′ 5′-CCGCCAGGACAAACCAGTAT-3′	94	No
***SDHA***	Succinate dehydrogenase complex, subunit A	NM_004168	5p15	5′-TGGGAACAAGAGGGCATCTG-3′ 5′-CCACCACTGCATCAAATTCATG-3′	86	Yes
***RPS13***	ribosomal protein S13	NM_001017	11p15.1	5′-CGAAAGCATCTTGAGAGGAACA-3′ 5′-TCGAGCCAAACGGTGAATC-3′	87	Yes
***HPRT1***	Hypoxanthine phosphoribosyl-transferase 1	NM_000194	Xq26.1	5′-TGACACTGGCAAAACAATGCA-3′ 5′-GGTCCTTTTCACCAGCAAGCT-3′	94	Yes
***18s***	18s rRNA	NT_167214.1	ChrUn*	5′-AGAAACGGCTACCACATCCA-3′ 5′-CACCAGACTTGCCCTCCA-3′	169	N/A
***B4GALT6***	UDP-Gal:βGlcNAcβ 1,4-galactosyl-transferase,polypeptide 6	NM_004775.3	18q12.1	5′-AGGAGGTCCCTATGGCACTAAC-3′ 5′-TCTCTACAGACAGGCCCATTAGTC-3′	89	No

* *Homo sapiens* unplaced genomic contig, GRCh37.p5.

The expression of the most stable genes was determined in human cell lines of normal surface epithelium of the ovary (n = 2) and of cancerous origin, including ovarian (n = 9), colon (n = 9), breast (n = 1), cervical (n = 1), uterine cancer (n = 1), and leukemia (n = 2).

qPCR was performed on the Stratagene Mx3005^®^ (Integrated Sciences Pty. Ltd, Chatswood, Australia) in 96-well microtitre plates (Bio-Rad Laboratories (Pacific) Pty Ltd, Gladesville, Australia). Optimal reaction conditions were obtained with 1× SsoFast™ EvaGreen^®^ Supermix with low ROX as the reference dye (Bio-Rad Laboratories (Pacific) Pty Ltd, Gladesville, Australia), 400nM specific sense primer, 400 nM specific antisense primer, RNase/DNase-free water, and cDNA template (previously isolated and reverse-transcribed RNA of 1 ng/well) up to final volume of 10 µl. Amplifications were performed starting with a 30 sec enzyme activation at 95°C, followed by 40 cycles of denaturation at 95°C for 5 sec, and then annealing/extension at 60°C for 30 sec. At the end of each run a melting curve analysis was performed from 65–95°C. All samples and negative controls were amplified in triplicate, and the obtained mean value was then used for further analysis. Cycle of quantification (Cq) values of >35 were excluded from further mathematical calculations. A “no template sample” (RNA from reverse transcription without reverse transcriptase) and a sample without RNA or cDNA were the negative controls.

### PCR Efficiency (E)

Comparability was ensured by investigating the qPCR efficiency on all reference genes on a randomly selected cell line RNA extract. To compare RNA transcript levels for the 12 putative reference genes, Cq values were generated directly at a specific threshold. The Cq is defined as the number of cycles needed for the fluorescence signal to reach a specific threshold of detection and is therefore inversely correlated to the input amount of total RNA [17]. To compare different qPCR runs performed on different days, plates and runs were adjusted to the threshold of Cq 0.1. Reverse transcribed cDNA was diluted from 100 ng to 1 pg of input RNA before RT in 10-fold dilutions for each reference gene in triplicate. Obtained fluorescence signals for definite RNA concentration were plotted and linear regression was performed to identify the best linear relationship representing the standard curve. The slope of the linear equation was applied to calculate the efficiency according to the equation E = (10^[−1/slope]^−1)×100.

### Data Analysis

Raw data including the melt and amplification curves obtained by MxPro- Mx3000P v4.10 (Integrated Sciences Pty. Ltd, Chatswood, Australia) were extracted to Microsoft^®^ Excel files (.xls), saved as Microsoft^®^ editor files (.txt), and then loaded for further data analysis into the open source statistical programming language R (http://CRAN.R-project.org/, version 2.13.2). For further modeling and analysis of RT-qPCR data, R package “qpcR” was used (http://cran.r-project.org/web/packages/qpcR/index.html). The Pearson correlation (r) was calculated to determine the association between applied algorithms.

To compare different algorithms for the selection of the most stable reference genes, we applied RefFinder (http://www.leonxie.com/referencegene.php), a web-based comprehensive tool. It uses the currently available algorithms geNorm [9], Normfinder [8], BestKeeper [10] and comparative ΔCt methods [11], and assigns an appropriate weight to an individual gene and calculates the geometric mean for overall ordering of all reference genes.

## Results

### Compliance of MIQE Guidelines for the Establishment of Reference Genes

**Figure 1:**
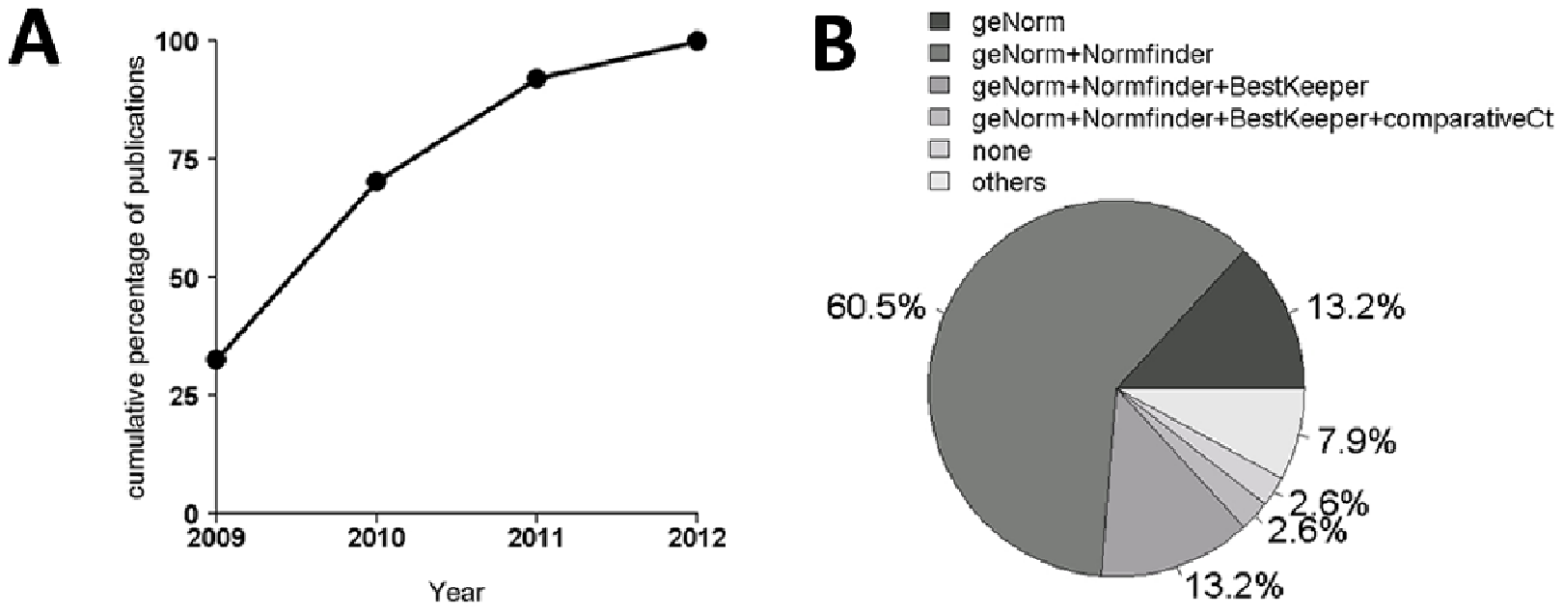
Number of RT-qPCR publications from 2009 to April 2012. (**A**). Line chart of all publications (n = 37) investigating the most stably expressed reference genes. (**B**) Percentage of algorithms used to identify reliable reference genes among all publications.

Since their introduction in 2009 [5], the research community is continuously accepting the MIQE guidelines for publications and consideration of scientific manuscripts using RT-qPCR. To study the acceptance of MIQE in more detail, we performed a literature review of publications proposing a set of putative reference genes (n≥5) in human samples for RT-qPCR. We identified 37 publications from January 2009 to April 2012, the number of which continuously increased each year (Figure 1A). Most of these studies investigated patient samples (63.2%), followed by primary and immortal cell lines (31.6%). A minor part (5.3%) examined tissue samples and cell lines together. RNA integrity was in most cases (68.4%) investigated with two independent methods such as spectrophotometry (90.9%) and RIN integrity number (Agilent Technologies, Inc., Santa Clara, CA, US). The amount of total RNA for reverse transcription or cDNA for qPCR reactions was reported in 78.9% of all publications. SYBR^®^ Green (57.9%), a fluorescent dsDNA binding dye, was more frequently used than TaqMan (26.3%), a fluorescently labeled target-specific probe. The qPCR efficiency for all investigated reference genes was provided in 68.4% of all publications, but only 28.9% provided details such as cDNA amount and the dilution range of standard curves. Finally, we investigated which of the known algorithms (geNorm, Normfinder, BestKeeper, comparative ΔCt) were applied to identify the most stably expressed reference genes. GeNorm and Normfinder algorithms together were used in most studies followed by geNorm alone and geNorm, Normfinder, and BestKeeper together (Figure 1B). Considering that only publications performing RT-qPCR for identifying stably expressed reference genes were included, these data demonstrate that essential information such as RNA integrity, the amount of total RNA in the reaction, qPCR efficiency, and cDNA amount is missing in a substantial fraction of these publications.

### Quality and Integrity of RNA Samples

Total RNA extracted from our set of cell lines was evaluated for quality and integrity. A detailed listing of the cell lines and the respective information on RNA amount, quality and integrity (absorbance ratios 260/230 nm and 260/280 nm, RNA integrity number RIN, and 28 s/18 s ratio) are presented as supplementary data (Table S2). We found that the absorbance ratios, averaged (mean ± standard deviation) over all 25 cell lines, were 1.97±0.23 (260/230 nm) and 2.11±0.04 (260/280 nm). We further found that the RIN ranged from 8.4 to 10, indicating a sufficient total RNA quality. The RIN algorithm assigns an RIN number score from 1 to 10, where a value of 10 represents completely intact RNA and a value of 1 represents degraded RNA. Moreover, the quality of total RNA was also confirmed by the ratio of 28 s/18 s ribosomal RNA, which ranged from 1.7 to 2.7.

The possible presence of contaminating DNA (Figure S1) in each qPCR experiment was investigated by amplification plots, melting curves, and 1% agarose gel electrophoresis. Reverse transcription negative control reactions confirmed the absence of contaminating DNA. Nevertheless, there were PCR amplifications in the negative controls of *PPIA*, *GUSB*, *RPII* and *TBP* in one or two replicates with Cq values >35. These values were substantially higher than those of the samples containing template of 1 ng cDNA, indicating a negligible amplification of contaminating DNA. No PCR amplifications in negative controls were detected for the other reference genes. These results indicate that our total RNA samples were of sufficient quality and largely free of contaminating DNA.

### qPCR Efficiency, Intra- and Inter-Assay Variability

The RT-qPCR efficiency of each primer set was determined by serial dilutions of cDNA template from the human ovarian surface epithelium cell line HOSE17-1 (Table 2). We used RNA from a randomly selected cell line rather than plasmids because RNA may cause potential non-negligible variation in reverse transcription [18], [19]. Based on the obtained mean Cq values for all dilutions in a logarithmic dilution series of cDNA, a standard curve was generated. The slope, intercept, qPCR amplification efficiency and correlation coefficients (R^2^) of each primer pair were calculated from the standard curve (Figure S2). The initial dilution range from 100 ng to 1 pg of input RNA before RT was adapted due to non-detectable amplicons at the lower range or saturation of qPCR reactions at higher cDNA amounts, both influencing efficiency in *RRN18S*, *RPII* and *B4GALT6* (Table 2). *GUSB* showed an optimal linear dilution range from 10 pg to 10 ng. PCR efficiency of studied reference genes ranged from 87.1% to 106.6%, slope from −3.680 to −3.157, intercept from 11.10 to 27.30 and R^2^ from 0.994 to 0.999.

**Table 2:** qPCR parameters providing the standard curve for each primer pair on 12 reference genes.

Gene	Slope	Intercept	Efficiency	R^2^	Dilution range
*HSPCB*	−3.250	20.09	103.1	0.998	1 pg-100 ng
*GAPDH*	−3.680	19.56	87.1	0.996	1 pg-100 ng
*YWHAZ*	−3.294	20.35	101.2	0.998	1 pg-100 ng
*SDHA*	−3.194	24.64	105.6	0.994	1 pg-100 ng
*RPII*	−3.157	23.72	107.4	0.998	10 pg-100 ng
*PPIA*	−3.251	20.09	103.1	0.998	1 pg-100 ng
*GUSB*	−3.213	25.81	104.7	0.999	10 pg-10 ng
*18sRNA*	−3.411	11.10	96.4	0.999	1 pg-10 ng
*RPS13*	−3.214	20.74	104.7	0.994	1 pg-100 ng
*HPRT1*	−3.173	23.66	106.6	0.997	1 pg-100 ng
*TBP*	−3.582	25.79	90.2	0.996	1 pg-100 ng
*B4GALT6*	−3.181	27.30	106.3	0.995	10 pg-100 ng

Relationship between Cq values and RNA concentration was calculated by linear regression to find a slope and intercept that predicts cDNA amounts and correlation coefficient (R^2^). QPCR efficiencies (E) were calculated based on the standard curve according to the equation [*E* = 10^(−1/slope)^−1]×100 and are expressed as a percentage.

To ensure a stable RNA transcript level, the influence of technical variability, intra- and inter-assay variation were investigated using the randomly selected *HSPCB* on RNA from SKOV3 cells. Inter-assay variation was determined by performing RT-qPCR with identical samples from five different days, revealing a coefficient of variation (CV) of 1.74%. The intra-assay variation was less than CV 0.3% using an amount of 0.5 ng and 2.0% using 5 pg of RNA. These data indicate that the technical variability is in an acceptable range and therefore is considered as negligible.

### Use of the Appropriate Reverse Transcriptase Setup

**Figure 2:**
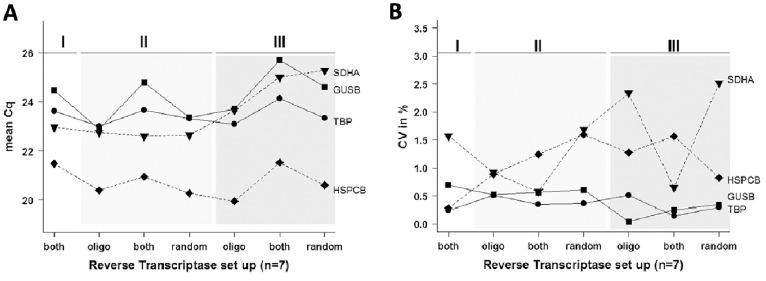
Different reverse transcription setups provided by three suppliers. (**A**) Random 6 mer were used in 2 and 5, oligo dT primer in 3 and 6, and both together in 1, 4 and 7 (abscissa); Cq on ordinate shows differences among the tested reverse transcriptase conditions. (**B**) Coefficient of variation (CV). RT suppliers indicated by roman numerals: I) BioRad, II) Takara, and III) Bioline. X-axis with different RT primers: oligo dT (oligo), random 6 mers (random), and both (both).

To study whether the origin (manufacturer) of the reverse transcriptases influences the quality of the reaction, four reference genes (*SDHA*, *HSPCB*, *GUSB* and *TBP*) were selected randomly and qPCR was performed after reverse transcription. To define the overall performance of all four selected reference genes, variations were calculated for the Cq and the CV values (n = 7). The variation for Cq ranged from a minimum of 1.14 (*TBP*) to a maximum of 2.83 (*GUSB*) (Figure 2A). The intra-assay variation within triplicates was highest in *SDHA* (CV = 2.51%) and lowest in *GUSB* (CV = 0.04%) (Figure 2B). These data indicate that the origin of the reverse transcriptase and different primer setups has only marginal influence in the performance of these four reference genes.

### Expression Stability of Candidate Reference Genes in Human Cell Lines

**Figure 3:**
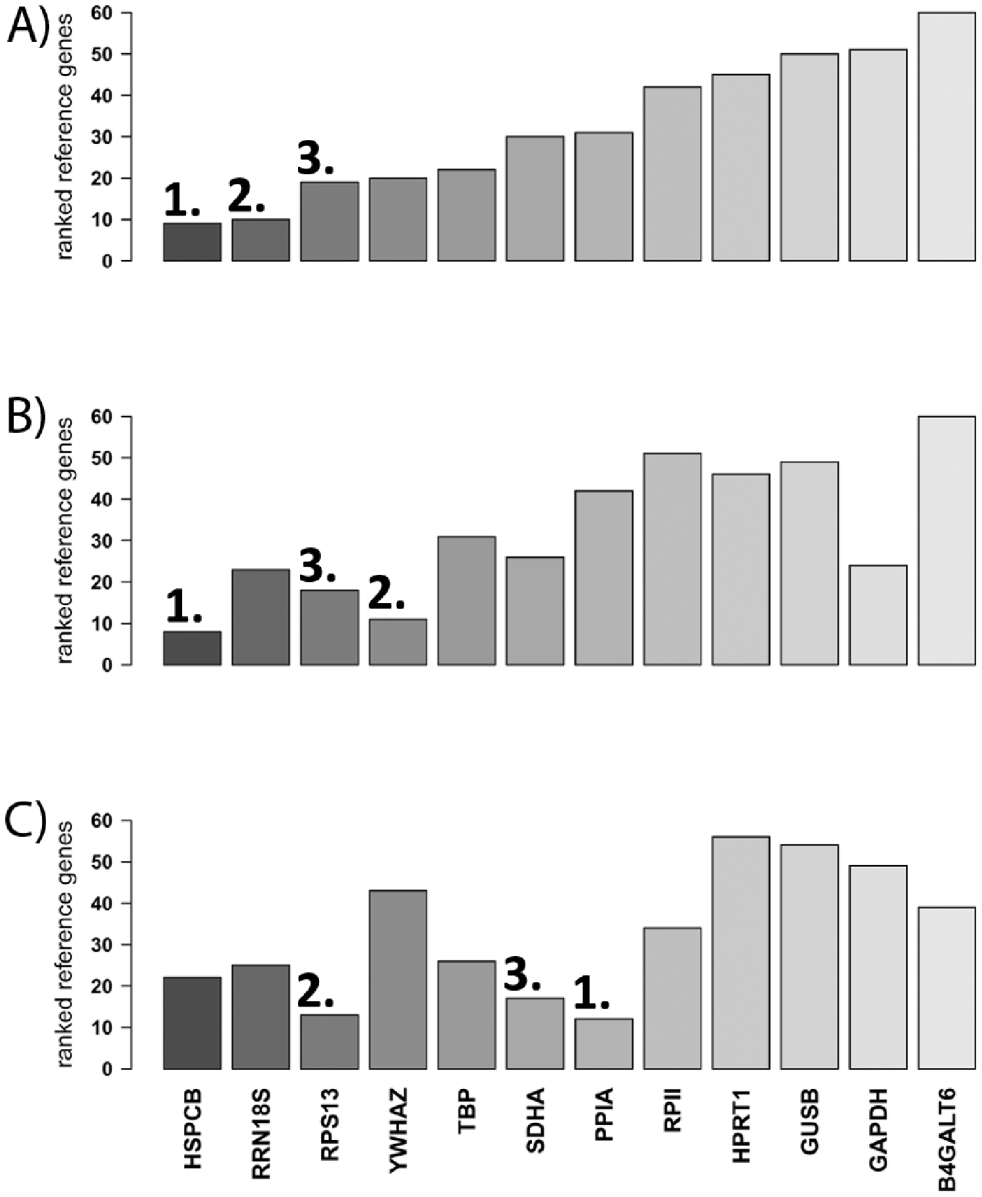
Bar graphs showing the most stably expressed genes calculated by rank sum of the 5 algorithms applied. (**A**) All investigated cell lines (n = 25), (**B**) colon cancer cell lines (n = 9), and (**C**) normal and ovarian cancer cell lines (n = 11). Numbers highlight the first three most stable reference genes in each experimental set up.

In order to identify the most stable reference genes across the tested normal and cancer cell lines, the expression stabilities of the reference genes were examined by performing the five algorithms (RefFinder, geNorm, BestKeeper, Normfinder, and comparative ΔCt) and ranking of the genes by each algorithm individually. These ranks were summed up, with the lowest rank sum representing the most stably expressed reference gene and the highest rank sum representing the least stably expressed reference gene. Across all investigated cell lines (Figure 3A) we identified *HSPCB*, *RRN18S* and *RPS13* as the 3 most stably expressed reference genes. *B4GALT6* was the least stable reference gene. In the subset of colon cancer cell lines *HSPCB*, YWHAZ, and *RPS13* were the 3 most stably expressed reference genes (Figure 3B). Moreover, in the subset of normal and ovarian cancer cell lines, the respective genes were *PPIA*, *RPS13* and *SDHA* (Figure 3C).

These results show that the most stably expressed reference genes vary among the different cell line sets, with only *RPS13* being present in all three cell line sets within the first 3 top reference genes. Interestingly, the most frequently used reference gene *GAPDH* was among the least stably expressed reference gene in our set up.

**Figure 4:**
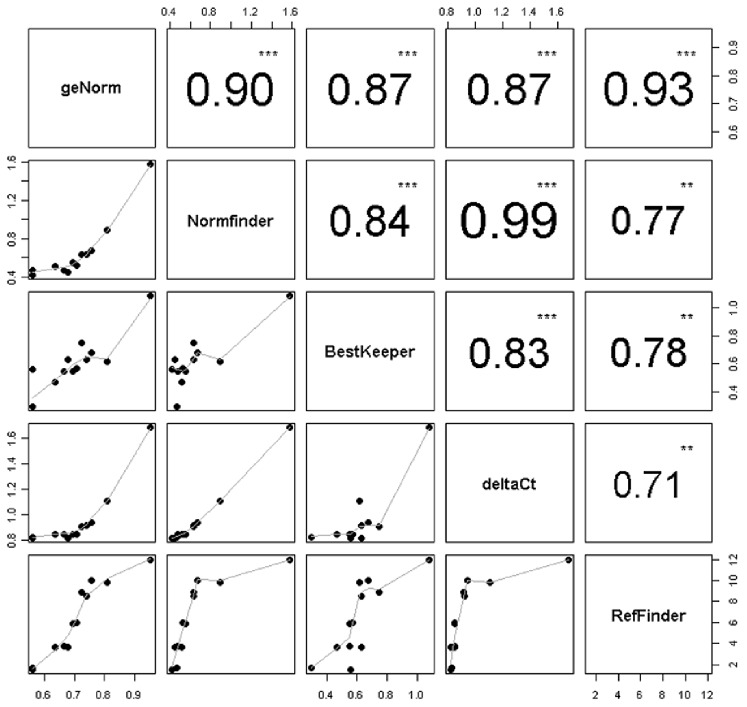
Correlation matrix visualizing reference genes ranked by five different stability tests (geNorm, Normfinder, BestKeeper, deltaCt and RefFinder). Absolute value of Pearson correlation and *p*-value indicated by asterisks (0***, 0.01**). Bottom of the scatter plots visualizes bivariate correlation among investigated stability tests including a fitted line.

We further investigated the correlation among the five applied algorithms that deliver the most stable genes. The correlation between all five stability tests was moderate to high among the applied algorithms. The highest correlation was observed between Normfinder and the comparative delta Ct method (r = 0.99) (Figure 4), indicating that all 12 reference genes were nearly identically ranked. The lowest correlation (r = 0.71) was between the delta Ct method and RefFinder, indicating a high discrepancy in ranking.

## Discussion

We 1) performed a literature review on the compliance of MIQE guidelines in the performance of RT-qPCR experiments identifying sets of reference genes, 2) assessed the performance of algorithms for the ranking of reference genes by their expression stability; and 3) identified a set of suitable and most reliable reference genes for our selection of human cancer cell lines of different origins.

Our literature review revealed that the compliance with the MIQE guidelines was only partial. Essential information [5] such as RNA integrity, the amount of total RNA in the reaction, cDNA amount, the dilution range for standard curves, and the efficiency of the qPCR is frequently missing in publications, which is not in compliance with the MIQE guidelines. Closest possible compliance with the guidelines for the performance of RT-qPCR as well as reporting this information in publications contributes to the improvement of the experimental study design and assures the reproducibility and reliability of the study results. Otherwise, minimal differences detected in targeted gene expression may be a potential result of variations in reference gene expression.

We have demonstrated that reverse transcriptases provided by different manufactures lead to variations in quantification cycles (Cq up to 3). Therefore it may be useful to consider different RTs and to test different primer sets, oligo dT or random 6 mers or both in combination prior to undertaking experiments. Our findings are also in concordance with previous studies where transcript yields varied up to 100-fold among different reverse transcriptases in a gene- dependent manner [20], [21]. We found in the literature and confirmed in our study that different RT priming strategies are crucial for quantitative measurement of gene expression [21]. Moreover, we observed in our own experiments that no RT is superior to other RTs and therefore no conclusion could be made for the choice of RT priming; that is, no decision can be made on which primer is better than the other.

The use of statistical algorithms for stability measurements was critically examined because all these algorithms are based on the assumption that none of the studied reference genes show systematic variation in the expression profile across the samples [22]. In addition, our literature review showed that in most studies only one or two algorithms have been applied. Our study revealed considerable variation in the correlation among the applied algorithms. Moreover, despite the relatively high correlation (r = 0.9) between the geNorm and the Normfinder algorithms, the application of these two algorithms delivered identical ranking in only 5 out of the investigated 12 reference genes. This presents a shortcoming that may lead to false selection of reference genes, which may have a negative impact on the quality and reliability of data. We therefore recommend that more than two algorithms should be applied for the selection of the reference genes.

We have utilized previously demonstrated and published primer pairs for detection of transcript levels of putative reference genes in a pool of normal and cancer cell lines. In general, nearly all reference genes performed in a suitable way and could be used for future studies using cell lines. In addition, based on results on intra- and inter-assay variability all investigated reference genes can be used for further investigations. Nevertheless, among all the cell lines tested we found that *HSPCB*, *RRN18S*, and *RPS13* are the most stably expressed genes suggesting their suitability as reference genes for future studies within this experimental set up. Investigations on colon and normal as well as ovarian cancer cell lines revealed expected discrepancies among selected reference genes and should be therefore considered in future studies. It demonstrates that reference genes have to be selected carefully for each experimental set up. Interestingly, *GAPDH* clearly performed with lower stability compared to other reference genes, suggesting reconsideration of the use of this gene in experiments. This is in concordance with the literature where variations in *GAPDH* expression are frequently observed throughout RT-qPCR experiments. Different disease stages can also have altered *GAPDH* gene expression. Certain experimental conditions may also influence the expression of *GAPDH*. These can be due to various factors: a) the selected primer pair is not the best choice for measuring gene expression, b) mRNA levels vary with cellular proliferation [23], c) influence of various factors on gene expression such as calcium and insulin [24], [25], d) altered expression among different tissue samples [9], [26], and f) contaminations due to the presence of pseudogenes [27].

Our findings are based on the use of five algorithms. Despite high correlations among those, which rank candidate reference genes, variations were still observed and can potentially influence our selection. However, we recommend using more than two algorithms for selecting the most stably expressed reference genes.

### Conclusion

For the establishment of a set of reference genes for target gene normalization in an experimental setup, we recommend, in concordance with the literature, the use of ideally 3 reference genes selected by at least 3 stability algorithms. It should be considered that ideal reference genes can vary with the set of cell lines under investigation and therefore these genes should be carefully selected for individual studies with the best possible compliance with the MIQE guidelines.

## Supporting Information

Figure S1 **Examples of the presence of genomic DNA.**
(PDF)

Figure S2 **Standard curve for all primer pairs.**
(PDF)

Table S1 **Culture conditions of cell lines.**
(PDF)

Table S2 **Quantity and integrity of total RNA.**
(PDF)
