# Supplementary material for: Careful Selection of Reference Genes Is Required for Reliable Performance of RT-qPCR in Human Normal and Cancer Cell Lines
Source: PLoS One. 2013 Mar 15;8(3):e59180. doi: 10.1371/journal.pone.0059180 (PMC3598660; doi:10.1371/journal.pone.0059180)
Supplement: Figure S1 — Examples of the presence of genomic DNA. (PDF) [file pone.0059180.s001.pdf]

Supporting Information 3

Figure S1 Examples of the presence of genomic DNA.

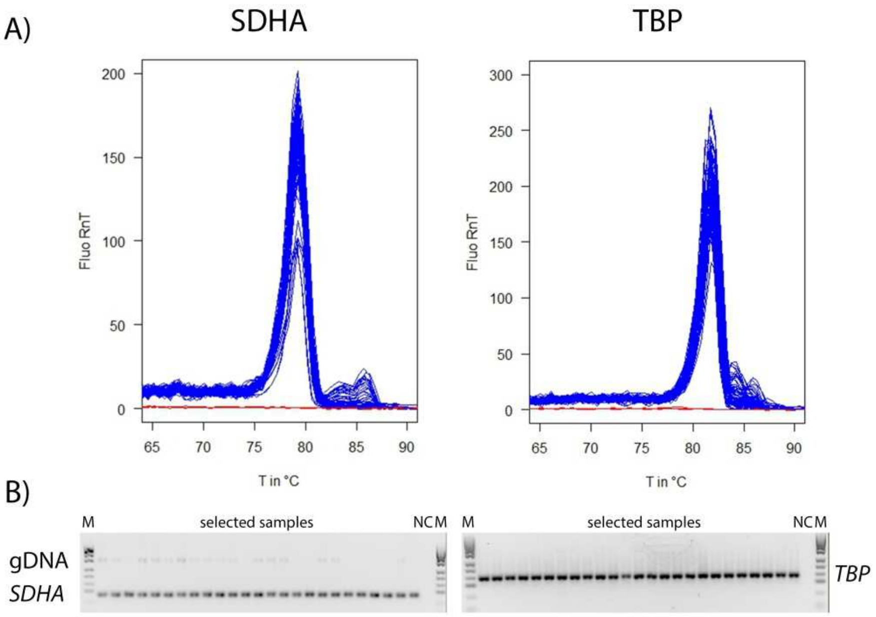

Melt curve (A) for *SDHA* and *TBP* demonstrates the presence of genomic DNA (gDNA) in few samples as confirmed by agarose gel electrophoresis. (B) Shows additional amplification of gDNA for *SDHA*. However, no additional amplification of gDNA was observed for *TBP* despite peaks in the melt curve. The melt curve (A) represents a 96-well plate including negative control (red) and standard curve. Negative control (NC), DNA ladder (M).
