# Supplementary figures and images for: Careful Selection of Reference Genes Is Required for Reliable Performance of RT-qPCR in Human Normal and Cancer Cell Lines
Source: PLoS One. 2013 Mar 15;8(3):e59180. doi: 10.1371/journal.pone.0059180 (PMC3598660; doi:10.1371/journal.pone.0059180)

## Supporting Information 4

**Figure S2** Standard curve for all primer pairs.

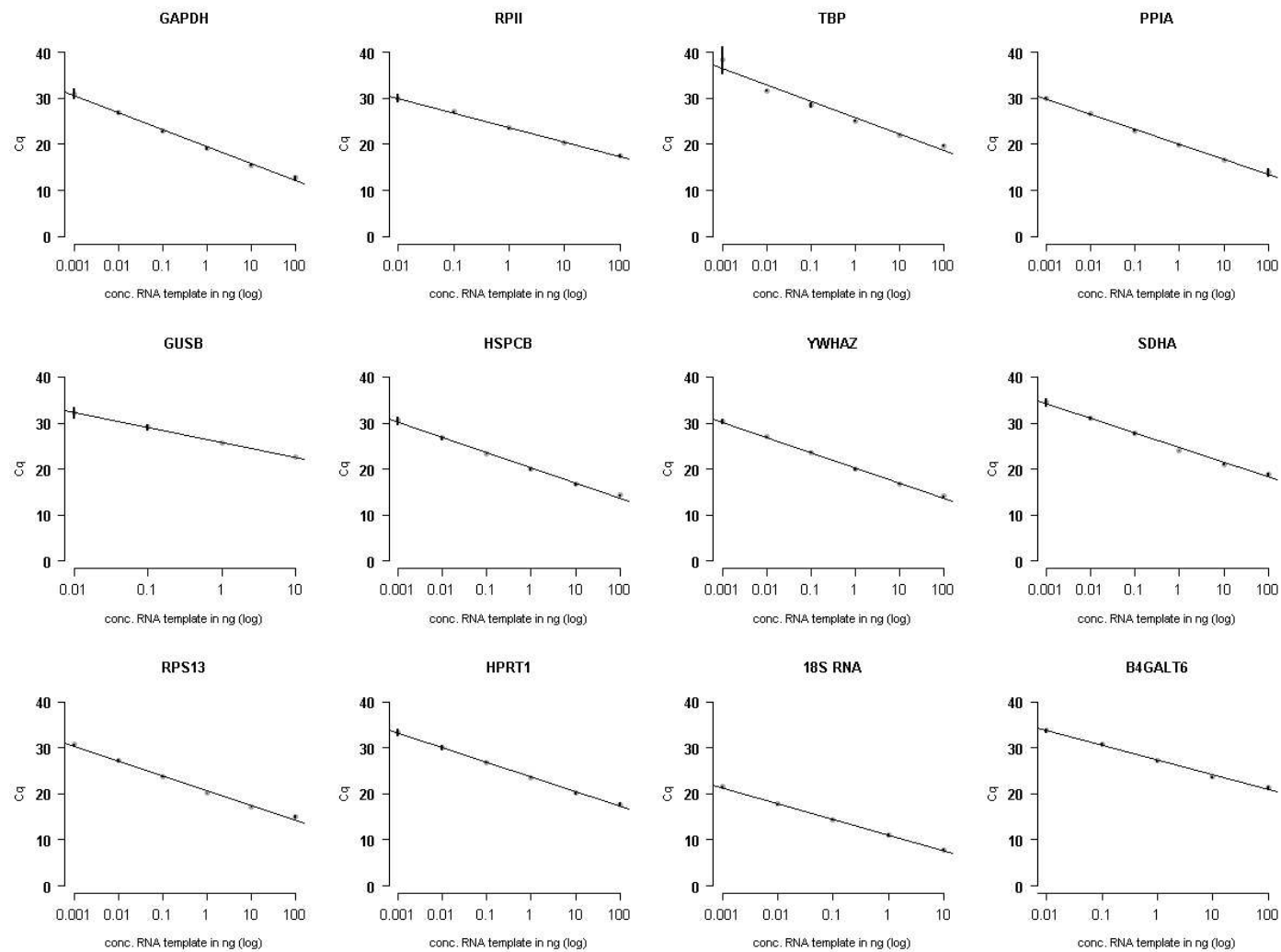

Supplement: Figure S2 — Standard curve for all primer pairs. (PDF) [file pone.0059180.s002.pdf]
