# Supplementary material for: Careful Selection of Reference Genes Is Required for Reliable Performance of RT-qPCR in Human Normal and Cancer Cell Lines
Source: PLoS One. 2013 Mar 15;8(3):e59180. doi: 10.1371/journal.pone.0059180 (PMC3598660; doi:10.1371/journal.pone.0059180)
Supplement: Table S1 — Culture conditions of cell lines. (PDF) [file pone.0059180.s003.pdf]

Supporting Information S1

Table S1 Culture conditions of cell lines.

Number	Cell Line	Origin	Media	FCS
1	HOSE17-1	normal ovary	HOSE media	10%
2	HOSE6-3	normal ovary	HOSE media	10%
3	TOV112D	ovarian	DMEM	10%
4	TOV21G	ovarian	DMEM	10%
5	EFO27	ovarian	RPMI	20%
6	SKOV3	ovarian	RPMI	10%
7	OVCAR3	ovarian	RPMI	10%
8	IGROV1	ovarian	RPMI	10%
9	A2780	ovarian	RPMI	10%
10	HCT15	colon	RPMI	10%
11	HCT116	colon	RPMI	10%
12	HT29	colon	DMEM	10%
13	Ls411	colon	RPMI	10%
14	S298	ovarian	DMEM	20%
15	SW480	colon	DMEM	10%
16	SW620	colon	DMEM	10%
17	HeLa	cervical	RPMI	10%
18	LS147T	colon	DMEM	10%
19	MCF-7	breast	RPMI	10%
20	2008	ovarian	DMEM	20%
21	RKO	colon	RPMI	10%
22	HEC59	uterine	Medium199	20%
23	Lim2142	colon	RPMI	10%
24	Jurkat	blood	RPMI	10%
25	THP1	blood	RPMI	10%

HOSE media consists of Medium 199:MCDB105 (50:50) mixed together. RPMI (Invitrogen #21870), DMEM (Invitrogen #11960), Medium 199 (Sigma #M2154), MCDB 105 (Sigma #M6395)
