# Supplementary material for: Careful Selection of Reference Genes Is Required for Reliable Performance of RT-qPCR in Human Normal and Cancer Cell Lines
Source: PLoS One. 2013 Mar 15;8(3):e59180. doi: 10.1371/journal.pone.0059180 (PMC3598660; doi:10.1371/journal.pone.0059180)
Supplement: Table S2 — Quantity and integrity of total RNA. (PDF) [file pone.0059180.s004.pdf]

Supporting Information S2

Table S2 Summarized results on RNA quality and integrity check in investigated cell line cultures.

Sample Number	Cell Line	RNA conc. (ng/µL)	260/280	260/230	RIN	28s/18s
1	HOSE17-1	178.27	2.08	2.11	10	2
2	HOSE6-3	339.41	2.09	1.95	10	2.2
3	TOV112D	722.58	2.16	1.69	10	1.9
4	TOV21G	607.33	2.11	2.2	10	2.1
5	EFO27	223.91	2.1	1.83	10	2.4
6	SKOV3	520.9	2.13	1.93	10	2
7	OVCAR3	244	2.08	2.13	10	2.1
8	IGROV1	302.63	2.09	2.16	10	2.3
9	A2780	757.08	2.1	2.21	10	2.2
10	HCT15	575.59	2.06	2.17	10	2.5
11	HCT116	552.86	2.15	1.51	9.5	2.7
12	HT29	913.34	2.16	2.07	10	2.2
13	Ls411	1173.87	2.14	2.2	10	2.4
14	S298	138.07	2.04	1.28	8.4	1.7
15	SW480	709.52	2.17	2.23	10	2.2
16	SW620	655.8	2.11	1.85	10	2.5
17	HeLa	961.1	2.11	1.92	10	2.4
18	LS147T	1084.61	2.11	2.13	10	2.3
19	MCF-7	553.74	2.1	2.13	10	2.3
20	2008	304.58	2.13	2.05	10	1.9
21	RKO	665.78	2.11	1.9	10	2.5
22	HEC59	782.74	2.14	2.11	10	2.2
23	Lim2142	282.24	2.17	2.13	10	2.2
24	Jurkat	650.21	2.13	1.84	10	2.3
25	THP1	268.51	2.12	1.54	10	2.6
